# Adipose Tissue Estrogen Receptor-Alpha Overexpression Ameliorates High-Fat Diet–Induced Adipose Tissue Inflammation

**DOI:** 10.1210/jendso/bvaf134

**Published:** 2025-08-19

**Authors:** Marion C Hope, Christian A Unger, M Chase Kettering, Cassidy E Socia, Ahmed K Aladhami, Barton C Rice, Darya S Niamira, Ben P Wiznitzer, Diego Altomare, William E Cotham, Reilly T Enos

**Affiliations:** Department of Pathology, Microbiology, and Immunology, University of South Carolina School of Medicine, Columbia, SC 29209, USA; Department of Pathology, Microbiology, and Immunology, University of South Carolina School of Medicine, Columbia, SC 29209, USA; Department of Pathology, Microbiology, and Immunology, University of South Carolina School of Medicine, Columbia, SC 29209, USA; Department of Pathology, Microbiology, and Immunology, University of South Carolina School of Medicine, Columbia, SC 29209, USA; Department of Pathology, Microbiology, and Immunology, University of South Carolina School of Medicine, Columbia, SC 29209, USA; Department of Pathology, Microbiology, and Immunology, University of South Carolina School of Medicine, Columbia, SC 29209, USA; Department of Pathology, Microbiology, and Immunology, University of South Carolina School of Medicine, Columbia, SC 29209, USA; Department of Pathology, Microbiology, and Immunology, University of South Carolina School of Medicine, Columbia, SC 29209, USA; Department of Drug Discovery and Biomedical Sciences, University of South Carolina College of Pharmacy, Columbia, SC 29208 USA; Department of Chemistry and Biochemistry, College of Arts and Science, University of South Carolina, Columbia, SC 29208, USA; Department of Pathology, Microbiology, and Immunology, University of South Carolina School of Medicine, Columbia, SC 29209, USA

**Keywords:** estrogen receptor-alpha, adipose tissue, estrogen, obesity, inflammation

## Abstract

**Context:**

We created an innovative mouse model that enables inducible overexpression of estrogen receptor-alpha (ERα), specifically in adipose tissue (Adipo-ERα↑).

**Objective:**

We aimed to investigate how elevated Adipo-ERα↑ influences the development of high-fat diet (HFD)-induced obesity in both male and female mice.

**Methods:**

Male and female Adipo-ERα↑ mice and littermate controls were fed a low-fat diet (LFD) or HFD for 13 weeks. Adipo-ERα↑ was induced at the initiation of dietary treatment. Body morphology and composition, hepatic lipid accumulation, glucose tolerance, fasting insulin concentrations, and adipose tissue mRNA profiling were assessed. Liquid chromatography–mass spectrometry was used to determine circulating and adipose tissue sex steroid content.

**Results:**

Adipo-ERα↑ significantly reduced adiposity and hepatic lipid accumulation in HFD-fed female mice but not in male mice. However, in both sexes, Adipo-ERα↑ greatly reduced adipose tissue inflammation characteristic of obesity. Despite these effects, Adipo-ERα↑ did not improve glucose tolerance or fasting insulin levels and did not affect circulating and adipose tissue sex steroid content.

**Conclusion:**

Adipo-ERα↑ elicits distinct sex-specific effects with respect to body composition and hepatic lipid accumulation, which are likely driven by variations in circulating and tissue estrogen levels. Nonetheless, despite differences in estrogen levels, Adipo-ERα↑ profoundly reduced obesity-linked adipose tissue inflammation in both sexes, providing further evidence that therapeutically targeting ERα may be beneficial for treating obesity-associated inflammation.

Obesity continues to be a global epidemic, with the prevalence of obesity in adults in the United States at 40% [1]. Although there are many defining features of the obese phenotype, 2 key characteristics are chronic low-grade inflammation centered in adipose tissue and impaired glucose metabolism [2]. It has previously been established that sex steroids, particularly 17β-estradiol, play a fundamental role in regulating inflammatory and metabolic processes in both males and females [3]. For example, menopause is linked to an increased risk of type 2 diabetes in women, and estrogen therapy has been shown to lower its incidence [4]. In males, aromatase inhibition (the enzyme that converts testosterone to estrogens) is associated with reduced insulin sensitivity [5]. Similar results have been shown in rodent models; ovariectomized mice are more susceptible to obesity development, including impaired glucose tolerance and increased adipose tissue inflammation, relative to gonadally intact mice [6, 7]. Additionally, 17β-estradiol administration protects male mice from obesity-related complications, including metabolic perturbations [8].

As scientists have begun to probe the tissue-specific action of 17β-estradiol signaling on metabolic and inflammatory processes, the role of 17β-estradiol signaling in adipose tissue has emerged. This has largely been achieved by manipulation of estrogen receptor-alpha (ERα), the receptor most responsible for eliciting the metabolic and anti-inflammatory effects of 17β-estradiol action [9-11]. Congenital deletion of adipose tissue ERα has highlighted the importance of adipose tissue 17β-estradiol signaling in regulating adipocyte metabolism and expansion, adiposity, glucose metabolism, and inflammation in both male and female mice [11-13]. Moreover, increasing adipose tissue 17β-estradiol content via aromatase overexpression has been shown to improve insulin sensitivity and reduce adipose tissue inflammation in male mice [14]. These insights are of clinical importance, as human studies have demonstrated that adipose tissue ERα expression is inversely related to adiposity and positively correlated with genes linked to metabolic health [12].

Although these studies have provided valuable insights into the role of adipose tissue 17β-estradiol signaling in metabolic and inflammatory processes, research gaps remain. For instance, while the anti-inflammatory and metabolic regulatory roles of 17β-estradiol via ERα in adipose tissue have been documented, the specific impact of ERα overexpression in adult adipose tissue remains unclear. Prior studies have relied on congenital transgenic models for ERα manipulation in adipose tissue, which do not allow for control over developmental compensation, complicating the understanding of 17β-estradiol signaling in adipose tissue after sexual maturity [11-13]. Additionally, no studies have explored gain-of-function 17β-estradiol signaling in adipose tissue through ERα overexpression. To address these gaps, we developed a novel mouse model that allows for inducible overexpression of adipose-specific ERα. Our novel mouse model allows for temporal control of ERα expression in adipose tissue. This enables the dissection of the role of ERα in adult mice, free from developmental compensation observed in congenital models. We used this model to investigate the effect of enhanced adipose tissue ERα on high-fat diet (HFD)-induced obesity. Our goal was to address the scientific gap in understanding the potential of targeting ERα overexpression in adipose tissue as a therapeutic approach to combat the inflammatory and metabolic complications associated with obesity.

## Methods

### Animals

**Figure 1.**
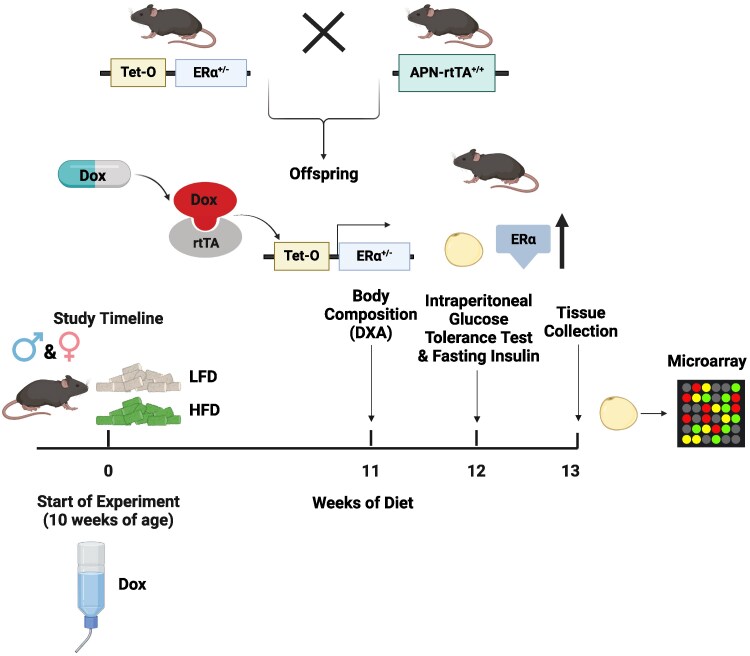
Schematic of the Adipo-ERα↑ model and experimental design (created with Biorender).

Adiponectin (APN)-rtTA^+/+^ [15] and tet-ERα^+/−^ mice [16] on a C57BL/6 background were bred to generate a novel mouse model that allows for inducible overexpression of ERα specifically in adipose tissue upon doxycycline (DOX) treatment (APN-rtTA^+/−^, tet-ERα^+/−^) (Fig. 1). We named this mouse model the Adipo-ERα↑ mouse model. Littermate controls (APN-rtTA^+/−^, tet-aromatase^−/−^) were used in all experiments. Inducible overexpression of ERα in Adipo-ERα↑ mice was achieved by providing 0.1 mg/mL DOX in drinking water throughout the duration of the study. To account for any potential side effects of DOX treatment, all groups received water supplemented with DOX. The drinking water was changed every week. Male and female mice were used in all experiments. We chose to initiate Adipo-ERα↑ at the initiation of dietary treatment to assess whether it could protect against the development of the obese phenotype.

### Diets

Male and female 10-week-old Adipo-ERα↑ and littermate control mice were randomly assigned to 1 of 4 groups of each sex: wild-type (WT) low-fat diet (LFD), Adipo-ERα↑ LFD, WT HFD, and Adipo-ERα↑ HFD groups. The diets were administered for 13 weeks. The LFD utilized in this experiment was the open-source, purified AIN-76A diet (3.79 kcal/g) (comprising 69%, 12%, and 19% of total kcals from carbohydrate, fat, and protein, respectively). The HFD (4.57 kcal/g) was a purified diet comprising 47%, 40%, and 13% of total kcals from carbohydrate, fat, and protein, respectively, with saturated fat making up 12% of total calories designed to mimic the standard American diet (BioServ, Frenchtown, NJ). Details and previous use of this diet are provided in Supplementary Table S1 [17]. The sample size for all experimental groups was n = 11-25 mice/group.

### Housing Conditions

Mice were housed, 4-5/cage, maintained on a 12:12-hour light-dark cycle in a low-stress environment (22.5 °C, 50% humidity, low noise), and provided food and water ad libitum. All experiments were performed in accordance with the American Association for Laboratory Animal Science, and the Institutional Animal Care and Usage Committee of the University of South Carolina approved all experiments.

### Body Weight and Body Composition

Body weights were monitored weekly throughout the study period. Body composition was assessed after 11 weeks of diet to use lean mass as the basis for glucose administration for glucose tolerance tests (GTTs). For this procedure, mice were briefly anesthetized via isoflurane inhalation, and lean mass, fat mass, and percent body fat were determined using dual-energy x-ray absorptiometry (DXA) (Lunar PIXImus, Madison, WI).

### Metabolic Assessment

The GTTs were performed after 12 weeks of dietary treatment. For these procedures, mice were fasted for 5 hours and glucose was administered intraperitoneally at 2 g/kg lean mass. A glucometer (Bayer Contour) was used to measure blood glucose concentrations (tail sampling) intermittently over a 2-hour period (0, 15, 30, 60, 90, and 120 minutes). Fasting plasma insulin concentrations were analyzed using a mouse insulin ELISA kit (Mercodia, Winston Salem, NC) (RRID: AB_2783837) according to the manufacturer's instructions. For circulating free fatty acid (FFA) assessment, blood was collected (tail sampling) after a 5-hour fast after 13 weeks of diet and serum was analyzed for FFAs (Wako, Fujifilm).

### Tissue Collection

At the end of each experiment, the mice were euthanized by isoflurane inhalation for tissue collection. Although all mice were euthanized in the same manner, it should be mentioned that isoflurane inhalation has been shown to elicit a stress response, which may impact sex steroid levels [18]. The time from when the initial “disturbance” was initiated to sample collection was approximately 3 minutes/animal. Blood was collected from the inferior vena cava using heparinized syringes and centrifuged to isolate plasma. The gonadal fat pad, skeletal muscle (gastrocnemius), and liver were removed and immediately snap-frozen in liquid nitrogen and stored at −80 °C until analysis. The mesentery and perirenal fat pads were excised and weighed, but they were not saved for gene expression analyses. In our unpublished observations, we have previously found that the gonadal fat pad typically exhibits the highest level of inflammation in response to HFD feeding when compared to LFD controls.

### Hepatic Lipid Accumulation and Histology

Lipids were isolated from the liver of each mouse using a modified Folch extraction method and quantified gravimetrically as previously described [19, 20]. A portion of the liver was formalin-fixed, embedded in paraffin blocks, sectioned, and stained with hematoxylin and eosin. Representative images were taken at 10× using a Nikon E600 microscope.

### Adipocyte Size and Adipose Tissue Histology

A portion of the adipose tissue was formalin-fixed, embedded in paraffin blocks, sectioned, and stained with hematoxylin and eosin. Adipose tissue images were taken using an Echo Rebel Microscope at 10×. The average adipocyte size of a minimum of 100 adipocytes per sample was determined. Only animals from the HFD groups were included in the adipose tissue histology. Representative images for publication purposes were taken at 10× using a Nikon E600 microscope.

### Microarray Experiments

An RNeasy Lipid Tissue Mini Kit (Qiagen, Valencia, CA, USA) including DNase treatment was used to isolate RNA from gonadal adipose tissue representing each of the HFD groups from both sexes. RNA quantity for n = 5-8/group was assessed using an Agilent 2100 Bioanalyzer and RNA Integrity Numbers (RIN) were >7.5. For gene expression experiments, total RNA was amplified and biotinylated using the GeneChip WT PLUS Reagent Kit (Thermo Fisher Scientific, Cat. No. 902930) according to the manufacturer's recommendations. Briefly, 100 ng of total RNA was reverse-transcribed into ds-cDNA using NNN random primers that also contained the T7 RNA polymerase promoter sequence. Subsequently, T7 RNA polymerase was added to the cDNA samples to amplify RNA molecules. Later, RNA was copied to ssDNA and subsequently removed using RNase H. Next, ssDNA molecules were fragmented and terminally labeled with biotin. Amplified/labeled samples were hybridized to Clariom S Mouse Arrays (Thermo Fisher Scientific, Cat. No. 902930) for 16 hours at 45 °C using GeneChip Hybridization Oven 645 (Thermo Fisher Scientific, Cat. No. 00-0331). Hybridized arrays were washed and stained using the kit mentioned above and GeneChip Fluidics Stations 450 (Thermo Fisher Scientific, Cat. No. 00-0079). Arrays were scanned with a GeneChip Scanner 3000 7G system (00-0218) using the Affymetrix GeneChip Command Console Scan Control software (version 4.0). Simultaneously, data were extracted from images, and the resulting probe cell intensity (CEL) files were imported into Transcriptome Analysis Console 4.0.2.15. CEL files were processed at the gene-level using Clariom_S_Mouse.r1.na36.mm10.a1.transcript.csv annotation file and the Signal Space Transformation—Robust Multichip Analysis (SST-RMA) algorithm to generate CHP files. After confirming data quality, phenotype-specific transcriptional responses were determined using one-way between-subject analysis of variance (ANOVA) with empirical Bayes correction. Differentially expressed genes with *P* values smaller than 0.05, and fold changes higher than 1.5 and lower than −1.5 were used for further bioinformatics analysis. Pathway analysis using the WikiPathway database, a component of TAC software, was used to determine the significantly affected physiological pathways.

### Quantitative Real-Time Polymerase Chain Reaction

For quantitative real-time polymerase chain reaction (qRT-PCR), an EZNA Total RNA Kit (Omega Bio-Tek, Norcross, GA, USA) was used to isolate RNA from gonadal adipose tissue, liver, and gastrocnemius. Bio-Rad reverse transcription reagents (Bio-Rad, Hercules, CA, USA) and Taqman probe assays (Thermo Fisher Scientific, Waltham, MA) were used to reverse transcribe and analyze the expression of the following genes in adipose tissue: ERα, ITGAX, MRC1, and CCL2. It is well-established that macrophages are the primary immune cells infiltrating adipose tissue in obesity [21]. As such we examined macrophage markers ITGAX and MRC1 and a macrophage-associated pro-inflammatory cytokine, CCL2, as validation genes of our microarray analysis. We have previously shown these genes to be upregulated resulting from the consumption of our custom HFD in previous investigations [19, 20, 22]. ERα mRNA expression was assessed in the skeletal muscle and liver to verify that ERα↑ was specific to the adipose tissue. Potential reference genes (HPRT, 18 seconds, GAPDH, β-Actin, HMBS, TBP, H2AFV, RPLPO, NONO, and B2M) were analyzed for stability using Qbase + software (Biogazelle, Ghent, Belgium) for each tissue analyzed. The optimal number of reference genes was determined using Qbase+, and the geometric mean of these genes was used as the normalization factor for each analysis. Gene expression was quantified using the ΔΔCT method and Qbase + software [23].

### Mass Spectrometry and Chromatography

Liquid chromatography–mass spectrometry (LC-MS) and LC-MS-MS were used to determine sex steroid concentrations in circulation (approximately 200 µL) and gonadal adipose tissue (approximately 300 mg) in HFD mice (LFD mice were not included due to limits in tissue amount) as previously described in detail [6, 20, 24]. In brief, prior to processing samples, internal standards (Sigma Aldrich, St. Louis, MO) for testosterone (−23.4-^13^C_3_), 17β-estradiol (D_5_), progesterone (D_9_), and androstenedione (−23.4-^13^C_3_) were added to each tissue and serum sample before tissue processing. Calibration curves utilizing certified reference material (testosterone, Sigma Aldrich, Catalog# T037; 17β-estradiol, Sigma Aldrich, Catalog# E-060; progesterone, Sigma Aldrich, Catalog# P-069; and androstenedione, Sigma Aldrich, Catalog# A-075) were used to determine the quantity of each steroid. Steroids were extracted from serum by vortexing with 500 mL of MS-grade methyl tert-butyl ether (MTBE) (×2) (Fisher Scientific, Waltham, MA, USA). The resulting upper phase was removed and placed in a glass vial, which was then dried using N_2_. The dried steroids were derivatized as previously described utilizing 1-methylimidazole-2-sulfonyl chloride [25]. For adipose tissue samples, tissues were weighed and homogenized in 1 mL of mass spectrometry grade acetonitrile (Fisher Scientific, Waltham, MA) for 1 to 2 minutes using a bead beater (Biospec Products, Bartlesville, OK) and 3.5-mm stainless steel UFO beads (Next Advance, Raymertown, NY). The samples were centrifuged for 5 minutes × 12 000 *g* and the resulting supernatant was removed and placed in glass vials. Subsequently, the tissue samples were resuspended in 1 mL of acetonitrile, homogenized, centrifuged, and the supernatant was collected a second time. Supernatants were dried using N_2_ and resuspended in 200 mM sodium acetate. MTBE was added to the sample for liquid–liquid extraction of the steroids (×2). The MTBE layer was removed and dried under N_2_ prior to the derivatization. Analyses were carried out on a Q Exactive HF-X hybrid quadrupole-orbitrap mass spectrometer with a Vanquish HPLC on the front end (Thermo Electron, Waltham, MA, USA) as previously described [24].

### Statistical Analysis

Data were analyzed using the commercially available statistical software Prism 10 (GraphPad Software, La Jolla, CA, USA). A two-way ANOVA (diet × genotype) followed by a Newman-Keuls post hoc test was used to assess differences between groups within each sex. For metabolic assessment over time, a two-way ANOVA (diet × genotype) followed by a Newman-Keuls post hoc test was performed within each time point. For sex steroid assessment, a two-way ANOVA (sex × genotype) followed by a Newman-Keuls post hoc test was used to assess differences between all HFD mice. For any two-way ANOVA, if a main effect for an interaction was not found to be statistically significant, we proceeded with multiple comparison analyses as suggested by Wei et al [26]. In their publication, Wei et al suggest performing multiple comparison analyses even when the interaction is not statistically significant, challenging the common misconception that comparisons among treatment means should be avoided under such conditions [26]. For any comparisons between 2 groups, a Student 2-tailed *t* test was used. Any statistical test that did not pass Spearman's test for heteroscedasticity or normality (D’Agostino-Pearson test) was log-transformed and then reanalyzed. Data are presented as mean ± SE, and the level of significance was set at *P* < .05. All F- and *P* values from all two-way ANOVAs are provided in Supplementary Materials [17].

## Results

### Adipose Tissue ERα↑ Is Specific to Adipose Tissue

**Figure 2.**
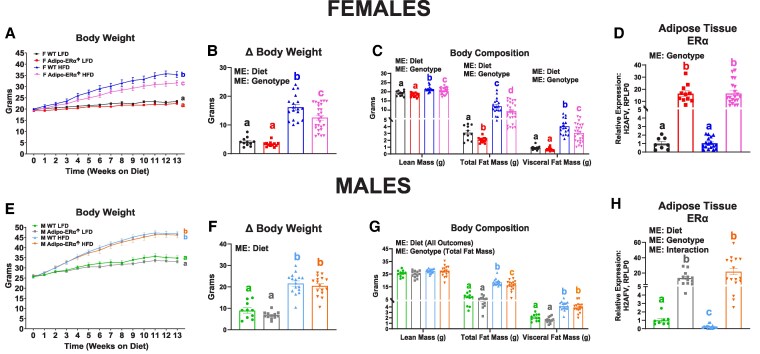
Adipo-ERα↑ affects body weight and body composition in a sex-dependent manner. (A&E) Body weight, (B&F) Δ body weight, and (C&G) body composition were assessed throughout and after 13 weeks of dietary treatment (low-fat diet [LFD] or high-fat diet [HFD]) in female and male wild-type (WT) and Adipo-ERα↑ mice (n = 11-25). (D&H) qRT-PCR confirmation in gonadal adipose tissue of ERα↑. Data are presented as mean ± SE. Bar graphs not sharing a common letter are significantly different from one another (*P* < .05).

Abbreviation: ME, main effect.

ERα overexpression was confirmed to be specific to adipose tissue (Supplementary Fig. S1A and S1B) [17] and was found to be overexpressed approximately 15-fold (Fig. 2D and 2H) (*P* < .05).

### Adipose Tissue ERα↑ Reduces Total Fat Mass in Both Sexes With a More Pronounced Effect Observed in Females

In female mice, adipose tissue ERα↑ resulted in decreased body weight gain in HFD-fed mice (Fig. 2A and 2B) (*P* < .05). Regarding body composition, lean mass increased with HFD consumption, although this increase was less pronounced in female Adipo-ERα↑ HFD mice (Fig. 2C). Both the LFD and HFD female Adipo-ERα↑ groups exhibited decreased total fat mass relative to their WT counterparts, with a significant decrease in visceral fat mass observed only in the Adipo-ERα↑ HFD group (Fig. 2C) (*P* < .05). This decrease in visceral fat mass was evident in the gonadal and retroperitoneal fat pads, but not the mesentery fat (Supplementary Fig. S2A-S2C) [17] (*P* < .05).

In male mice, adipose tissue ERα↑ had no effect on body weight gain relative to WT controls (Fig. 2E and 2F) (*P* < .05). However, total fat mass was significantly reduced in Adipo-ERα↑ HFD mice relative to their WT counterparts (Fig. 2G) (*P* < .05). Adipo-ERα↑ had no impact on total visceral fat mass accumulation in both LFD and HFD settings. However, with respect to individual visceral fat pad weights, the HFD Adipo-ERα↑ mice exhibited a reduced retroperitoneal fat pad weight relative to HFD WT mice, which was not observed in the gonadal and mesentery fat pads (Supplementary Fig. S2D-S2F) [17] (*P* < .05). HFD consumption reduced adipose tissue ERα expression in WT mice, which was not observed in female mice (Fig. 2H) (*P* < .05).

### Sex, But Not Adipo-ERα↑, Affects Serum and Gonadal Sex Steroid Content

There was a main effect of sex on steroid content in the serum and gonadal adipose tissue of HFD mice (Table 1). 17β-estradiol and progesterone levels were increased in the serum and gonadal adipose tissue of female mice, irrespective of genotype (*P* < .05). Conversely, testosterone and androstenedione levels increased in the serum and gonadal adipose tissue of male mice (*P* < .05). There was no effect of the Adipo-ERα↑ genotype to impact any of the steroids assessed in serum or gonadal adipose tissue. We did not determine if Adipo-ERα↑ impacted the normal cyclicity of the estrous cycle in the female mice. However, given the significant range of circulating and tissue sex steroids within the Adipo-ERα↑ female mice, it is our belief that these mice continued to cycle.

**Table 1.** Sex, but not Adipo-ERα↑, affects circulating and adipose tissue sex steroid levels

	Serum				
	F WT HFD	F Adipo-ERα↑ HFD	M WT HFD	M Adipo-ERα↑ HFD	Main effect
17β-Estradiol (pg/mL)	3.6 (±1.0)^a^	3.4 (±0.9)^a^	0.13 (±0.1)^b^	0.08 (±0.1)^b^	Sex
Testosterone (pg/mL)	24.9 (±5.8)^a^	17.2 (±2.4)^a^	4670 (±1666.0)^b^	1811 (±914.0)^b^	Sex
Androstenedione (pg/mL)	20.5 (±3.7)^a^	15.1 (±1.6)^a^	145 (±42.2)^b^	62.9 (±27.3)^b^	Sex
Progesterone (ng/mL)	6.04 (±2.2)^a^	7.4 (±2.2)^a^	1.54 (±0.3)^b^	2.00 (±0.5)^b^	Sex

	Gonadal adipose tissue				
	F WT HFD	F Adipo-ERα↑ HFD	M WT HFD	M Adipo-ERα↑ HFD	Main effect
17β-Estradiol (pg/g)	15.5 (±11.8)	13.3 (±5.8)	0.86 (±0.6)	2.23 (±2.9)	Sex
Testosterone (pg/g)	39.2 (±7.4)^a^	32.0 (±5.2)^a^	11 199 (±3465.0)^b^	7964 (±3292.0)^b^	Sex
Androstenedione (pg/g)	657 (±147.0)^a^	445 (±84.0)^a^	2194 (±485.0)^b^	2387 (±643.0)^b^	Sex
Progesterone (ng/g)	298 (±95.4)^a^	191 (±48.2)^a^	3.0 (±0.3)^b^	3.7 (±0.6)^b^	Sex

Serum (n = 15-21/group) and gonadal adipose tissue (n = 8-12/group) sex steroid content were analyzed. Data are presented as mean ± SE.

Abbreviations: ERα, estrogen receptor-alpha; HFD, high-fat diet; WT, wild-type.

^a, b^ Letters that do not match are statistically different from one another (*P* < .05).

### Adipose Tissue Inflammation Is Significantly Lower in Both Male and Female HFD-Fed Adipo-ERα↑ Mice

**Figure 3.**
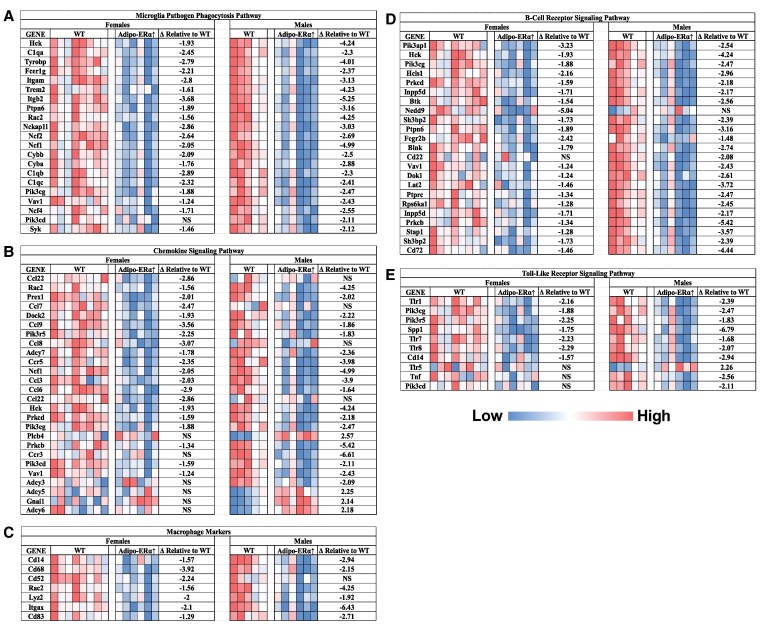
Microarray analysis shows Adipo-ERα↑ blunts HFD-induced adipose tissue inflammation in both sexes. A microarray analysis was performed in the gonadal adipose tissue of female and male HFD WT and Adipo-ERα↑ mice (n = 5-8). Multiple genes involved in the following signaling pathways were found to be statistically different (*P* < .05): (A) microglial pathogen phagocytosis pathway, (B) chemokine signaling pathway, (C) macrophage markers, (D) b-cell receptor signaling pathway, and (E) toll-like receptor signaling pathway.

Abbreviation: NS, not significant.

Microarray experiments performed on the adipose tissue of HFD mice showed that Adipo-ERα↑ resulted in decreased expression of genes linked to various inflammatory pathways in both male and female mice relative to their WT HFD littermate controls (Fig. 3A-3E and Supplementary Table S2) [17] (*P* < .05). We highlighted the pathways and genes found to be modulated within these pathways in Fig. 3A-3E. Only individual genes that were found to be ±2.0-fold changed in either male or female Adipo-ERα↑mice relative to their WT controls were included. These pathways include the “microglia pathogen phagocytosis pathway” (Fig. 3A), the “chemokine signaling pathway” (Fig. 3B), “macrophage markers” (Fig. 3C), “B-cell receptor signaling pathway” (Fig. 3D), and the “toll-like receptor signaling pathway” (Fig. 3F). In addition to these pathways, we identified several other pathways that were either similarly or differently affected by Adipo-ERα↑ in each sex compared to their WT HFD littermate controls (Supplementary Table S2) [17] (*P* < .05). Of interest was the finding that both males and females displayed a significant reduction in the expression of genes regulating the matrix metalloproteinases, whereas only males exhibited an increase in the expression of genes known to regulate adipogenesis (Supplementary Table S2) [17] (*P* < .05). While most of the statistically significant pathways were common between male and female Adipo-ERα↑mice, male Adipo-ERα↑ mice exhibited a greater number of pathways with statistically significant changes compared to females.

**Figure 4.**
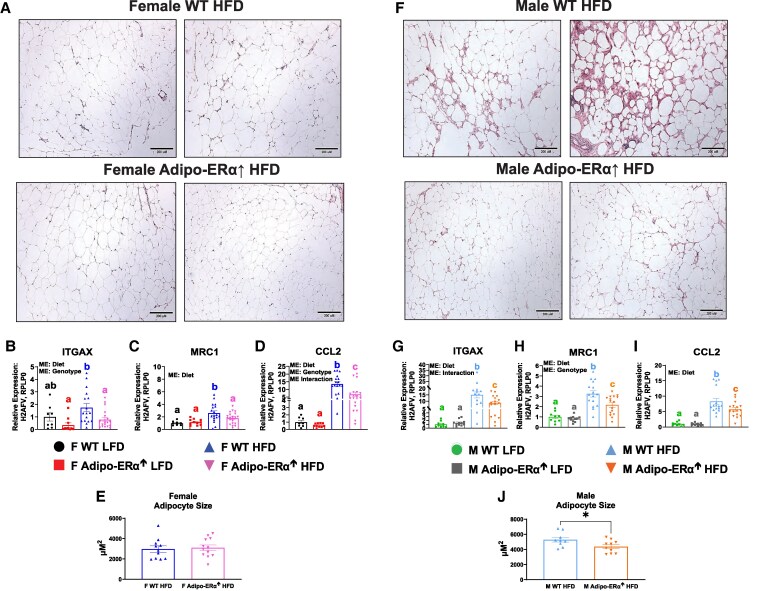
Adipose inflammation is reduced in HFD Adipo-ERα↑ mice, irrespective of sex. The microarray results were confirmed by histology and qRT-PCR. (A&F) Representative H&E staining of adipose tissue (10× magnification), adipose tissue gene expression of inflammatory markers (B&G) ITGAX, (C&H) MRC1, (D&I) CCL2, and (E&J) mean adipocyte size. Bar graphs not sharing a common letter are significantly different from one another (*P* < .05). * = *P* < .05.

Abbreviation: ME, main effect.

Histological analysis revealed that WT HFD mice, regardless of sex, exhibited greater adipose tissue inflammation than their Adipo-ERα↑ HFD counterparts (Fig. 4A and F). This finding was further confirmed by qRT-PCR validation of the microarray data (Fig. 4B-4D, 4G-4I). Both female and male HFD Adipo-ERα↑ mice presented decreased expression of the pro-inflammatory macrophage marker, ITGAX (Fig. 4B and 4G, *respectively*), the anti-inflammatory macrophage marker, MRC1 (Fig. 4C and 4H, *respectively*), which is thought to be increased to counteract the heightened pro-inflammatory environment, and the pro-inflammatory chemokine, CCL2 (Fig. 4D and 4I, *respectively*) (*P* < .05).

The average adipocyte size was unchanged between female WT HFD and female Adipo-ERα↑ mice (Fig. 4E). In males, however, the average adipocyte size was found to be significantly smaller in the HFD Adipo-ERα↑ mice than in littermate controls (Fig. 4F) (*P* < .05).

### Liver Weight and Hepatic Lipid Accumulation Is Decreased in HFD-Fed Female Adipo-ERα↑ Mice

**Figure 5.**
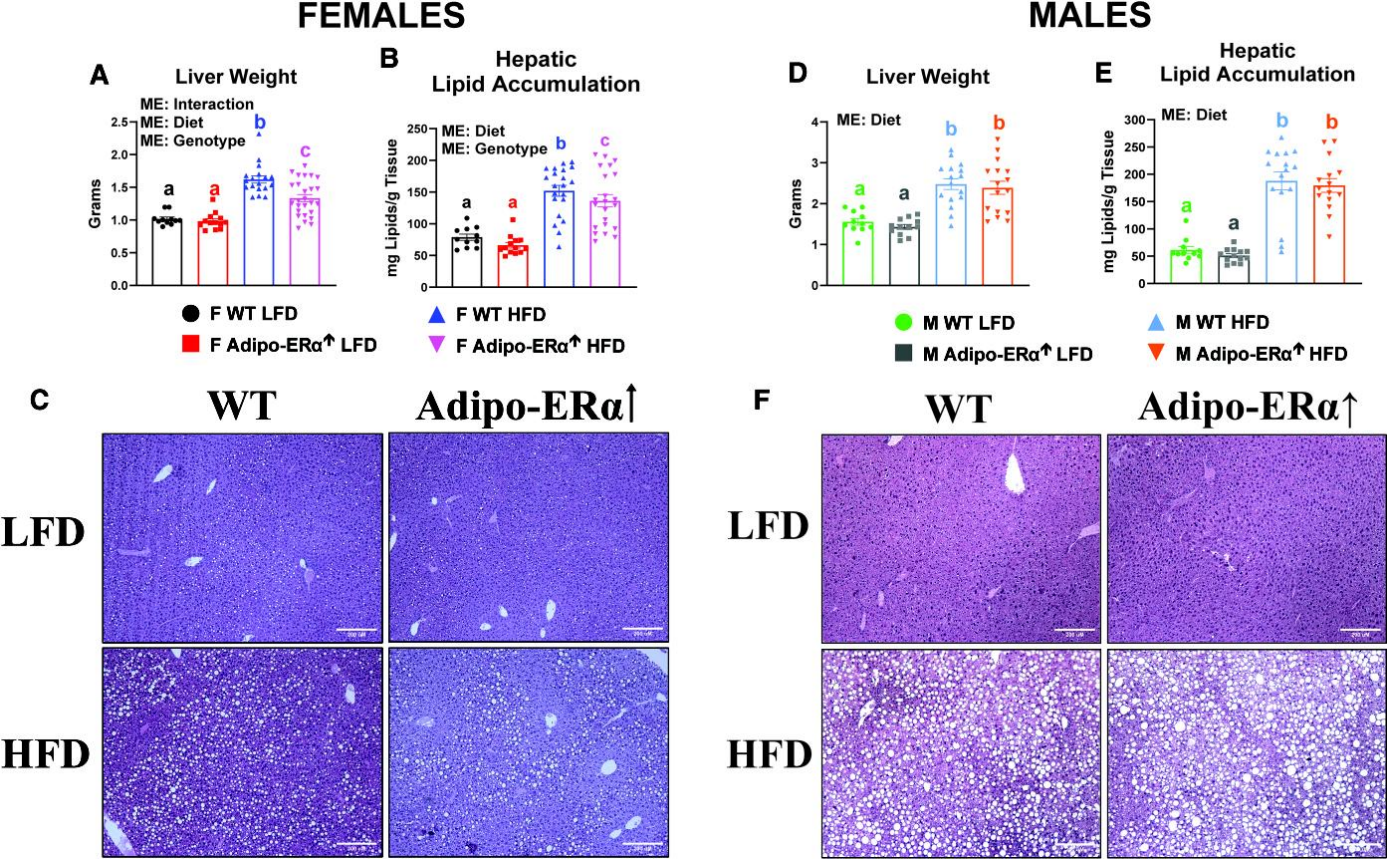
Adipo-ERα↑ mitigates HFD-induced lipid accumulation in female mice. (A&D) Liver weight and (B&E) hepatic lipid accumulation were assessed after 13 weeks of dietary treatment (low-fat diet [LFD] or high-fat diet [HFD]), respectively, in male and female wild-type (WT) and Adipo-ERα↑ mice (n = 11-25). (C&F) Representative hepatic hematoxylin and eosin images (10× magnification). Data is presented as mean ± SE. Bar graphs not sharing a common letter are significantly different from one another (*P* < .05).

Abbreviation: ME, main effect.

In female mice, HFD consumption increased liver weight and hepatic lipid accumulation, which were mitigated in Adipo-ERα↑ mice (Fig. 5A-5C) (*P* < .05). In male mice, although there was a HFD effect to increase liver weight and hepatic lipid accumulation, Adipo-ERα↑ had no effect on liver weight or hepatic lipid accumulation (Fig. 5D-5F).

### Glucose Tolerance, Fasting Insulin Concentrations, and Serum FFAs Are Not Affected by Adipo-ERα↑

**Figure 6.**
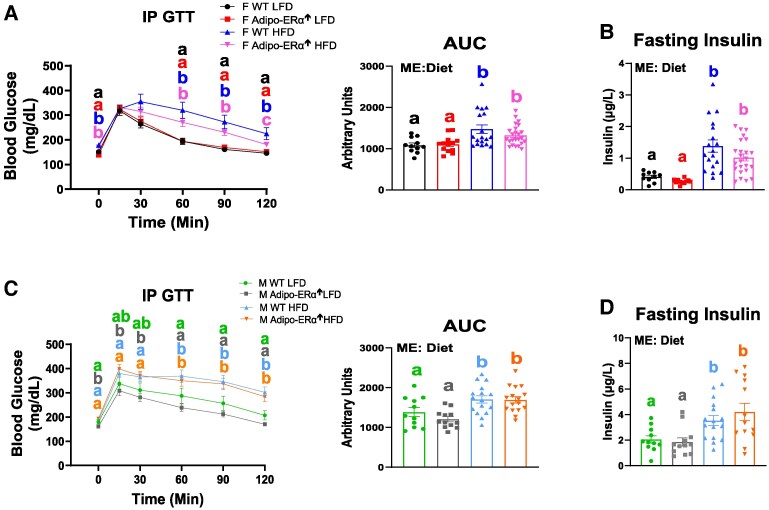
Adipo-ERα↑ does not hinder impairments to glucose metabolism. Glucose metabolism was assessed by an (A&C) intraperitoneal glucose tolerance test (IP GTT) and (B&D) fasting insulin levels. Bar graphs not sharing a common letter are significantly different from one another (n = 11-25) (*P* < .05).

Abbreviations: AUC, area under the curve; ME, main effect.

In both sexes, HFD consumption impaired glucose tolerance and induced hyperinsulinemia, irrespective of genotype (Fig. 6A-6D) (*P* < .05). No difference in serum FFAs was found across any of the groups within each sex (Supplementary Fig. S3) [17].

## Discussion

17β-estradiol is known to be a key regulator of metabolic health and inflammatory processes. Recent studies have begun to explore the tissue-specific effects of 17β-estradiol action. Here, we demonstrate that enhancing 17β-estradiol signaling by inducing ERα overexpression in adipose tissue can reduce obesity-associated adipose tissue inflammation in both sexes, which can occur independent of body weight changes. Notably, in females, Adipo-ERα↑ reduced body weight, visceral fat mass, and hepatic lipid accumulation in the setting of a HFD, which was not observed in males.

Our findings are consistent with other studies utilizing “loss of function” approaches involving congenital deletion or viral-mediated knockdown of ERα to interrogate the role of adipose tissue 17β-estradiol signaling on metabolic and inflammatory outcomes [11]. For instance, using the Cre-lox system driven by the adiponectin promoter, Davis et al showed that adipocyte deletion of ERα in the setting of a chow diet increased body weight and fat mass in female mice, but not in male mice [11]. Additionally, the authors discovered that adipose tissue inflammation and fibrosis were exacerbated in ERα adipose tissue–deficient mice and in fat pads in which ERα knockdown was achieved, with this effect being more pronounced in male mice [11]. A key difference between our study and that of Davis et al is that their observed phenotype emerged under LFD (chow) conditions, whereas we did not observe a similarly pronounced effect of Adipo-ERα↑ in our LFD groups. This discrepancy may be explained by the fact that the LFD used in our study is a well-established, purified, open-source diet with a consistent nutritional profile, unlike the “chow” diet used by Davis et al. However, what is most likely to account for the differences between the 2 studies is that our study focuses on a gain-of-function approach, augmenting adipose tissue ERα, whereas Davis et al employed a loss-of-function approach. Consequently, the differences in results under LFD conditions may stem from the fact that a lack of ERα likely disrupts adipose tissue physiology, while Adipo-ERα↑ in an “unchallenged” LFD setting may not as drastically impact adipose tissue morphology and metabolism.

It is evident from both our work and that of Davis et al that a sexual dimorphism exists with respect to the impact of adipose tissue ERα on body weight. Consistent with Davis et al, we found that females were more responsive to adipose tissue ERα manipulation in terms of body weight changes than males. We hypothesize that this is a result of different circulating and adipose tissue 17β-estradiol levels between the sexes. We have previously characterized sex steroid fluctuations in various tissues throughout the murine estrous cycle [24]. In this current investigation, we also examined adipose tissue and circulating sex steroids. While we did not observe any effect of Adipo-ERα↑ to impact sex steroid content, we did find an effect of sex to influence circulating and adipose tissue sex steroid levels. Given that females naturally have higher 17β-estradiol levels than males, changes in ERα expression, either loss or gain, likely have a more pronounced effect on body weight. The hypothesis that the quantity of adipose tissue 17β-estradiol content can affect body weight is also supported by the work of Ohlsson et al, who overexpressed aromatase in adipose tissue using the aP2 promoter and found that increased 17β-estradiol content in adipose tissue was associated with decreased body weight in male mice [14]. However, no changes in body weight were observed in female mice overexpressing aromatase, likely because they did not exhibit elevated adipose tissue 17β-estradiol levels [14]. This sex-dependent effect may be attributed to the higher basal testosterone in male mice, which is readily converted into 17β-estradiol. We observed similar substrate-dependent effects when examining aromatase overexpression in the skeletal muscle in both sexes [6, 20].

This raises the question of what specific processes in the 17β-estradiol-ERα signaling cascade in adipose tissue regulates body weight changes? A limitation of our study is that we did not directly monitor food intake or perform indirect calorimetry experiments to determine the factors that may explain body weight differences. However, we believe that the reduced body weight we observed is not likely due to changes in food intake, as Davis et al also observed a change in body weight with adipocyte ERα deletion with no change in food intake [11]. Thus, the body weight difference is likely driven by variations in energy expenditure. Previous research by Zhou et al showed that ERα regulates adipocyte metabolism and mitochondrial remodeling via Polg1, which can also act in brown adipose tissue to control energy homeostasis via UCP1 [12]. They found that brown fat–deficient ERα mice exhibited increased weight gain relative to controls [12]. In support of this, Santos et al found that activation of ERα induces beiging of adipocytes [27]. As the Adiponectin-rtTA system is not specific to white adipose tissue, ERα overexpression in brown adipose tissue may have led to increased energy expenditure, contributing to the reduced body weight phenotype observed in our study. However, it is important to note that we did not observe changes in Polg1 expression in our microarray data, nor did we detect alterations in mitochondrial pathways resulting from ERα↑, in contrast to Zhou's findings in their ERα-deficient models. Zhou et al used congenital models of adipocyte ERα deletion, whereas we employed a postmaturation inducible overexpression model. Thus, ERα may play a more significant role in regulating the mitochondrial dynamics of adipose tissue during development than during adulthood. Furthermore, augmentation of adipose tissue ERα likely produces a phenotype different from that of a loss-of-function scenario. Future studies using inducible ERα deletion models are necessary to explore this further.

An additional research question of interest is whether 17β-estradiol directly regulates inflammation in the setting of obesity or whether the reduction in inflammation is a consequence of enhanced adipose tissue storage capacity, manifested as adipocyte hypertrophy or adipogenesis. Unfortunately, our study design cannot answer this question. We did observe that male HFD Adipo-ERα↑ mice exhibited a decrease in adipocyte size, whereas no changes to adipocyte size were observed in female HFD Adipo-ERα↑ mice. Previous work by others provides evidence that increased 17β-estradiol content in adipose tissue and 17β-estradiol cycling induces adipogenesis [14, 28]. In support of this, the adipogenesis (26 genes# and 7 genes↓) and matrix metalloproteinase (4 genes# and 10 genes↓) pathways were altered by Adipo-ERα↑ (the adipogenesis pathway only in male mice). The expression of matrix metalloproteinases has been shown to change during adipose tissue remodeling [29, 30]. However, if adipogenesis was a major factor in the outcomes of Adipo-ERα↑, we would have expected to see a reduction in ectopic lipid accumulation, but we did not observe this in male Adipo-ERα↑ mice, as evidenced by the unchanged hepatic lipid accumulation. We believe that the reduced hepatic lipid accumulation observed in the female HFD Adipo-ERα↑ mice is due to a reduction in adiposity rather than a direct effect of increased adipogenesis. Future studies focusing on how Adipo-ERα↑ regulates adipocyte morphology are needed.

It is noteworthy that we did not observe any beneficial effects of Adipo-ERα↑ on glucose tolerance or hyperinsulinemia. This was unexpected, given the significant reduction in adipose tissue inflammation in both sexes as well as the notable decrease in body weight and hepatic lipid accumulation in female HFD Adipo-ERα↑ mice. Similar findings were reported by Davis et al, who, despite observing body weight gain in female adipocyte ERα-knockout (KO) mice, did not see improvements in glucose tolerance [11]. Conversely, male adipocyte ERα KO mice showed no change in body weight but exhibited significant increases in adipose tissue inflammation and disrupted glucose metabolism [11]. Previous studies have identified a clear association between obesity-associated adipose tissue inflammation and impaired glucose metabolism [31]. However, the causal relationship between adipose tissue inflammation and impaired glucose metabolism in obesity remains a subject of debate [31, 32]. Mechanistic research has shown that drastic suppression of adipose tissue inflammation promotes insulin resistance despite beneficial effects on weight gain [33]. The authors suggested that adipose tissue inflammation is an adaptive response to hypoxia and is necessary for proper adipose tissue expansion [33]. Additionally, time-course experiments have shown that insulin resistance precedes adipose tissue inflammation and is associated with specific lipid species [34]. We have consistently demonstrated that reductions in adipose tissue inflammation do not always correlate with improvements or impairments in glucose metabolism or hyperinsulinemia [6, 19]. This suggests that the degree to which adipose tissue inflammation is suppressed differentially affects glucose metabolism. Moreover, reducing inflammation, even without affecting glucose metabolism, may be beneficial because obesity-related inflammation is associated with various comorbidities, including cancer [35, 36]. We should also point out that our assessment of glucose tolerance was limited to an intraperitoneal glucose tolerance test (IP GTT), which, although informative and is an indicator of glucose handling, may not reflect the more physiologically relevant oral glucose tolerance test [37]. Additionally, we did not directly assess insulin action in these mice. Nonetheless, in support of the absence of improvements to glucose tolerance, Adipo-ERα↑ did not favorably affect hyperinsulinemia in either sex. Perhaps, if we had extended the duration of the study resulting in increased adiposity, we may have been able to observe beneficial effects of Adipo-ERα↑ on glucose metabolism. Of note is that we did not observe a difference in circulating FFA levels for either sex in the Adipo-ERα↑ despite changes to fat mass or adipocyte size. One may expect that the ERα overexpression would lead to increased FFA levels indicative of enhanced lipolysis. However, it should be made clear that a single measurement of circulating FFAs does not capture the rate of FFA flux. Therefore, even though the groups show similar circulating FFA concentrations, there could be differences in FFA flux rates, suggesting differences in the rate of lipolysis.

It must be highlighted that the importance of 17β-estradiol signaling in regulating metabolic and inflammatory outcomes in males is understudied but is starting to be appreciated. Growing evidence suggests that 17β-estradiol plays an important role in males, as it does in females. For instance, male whole-body ERα KO mice display increased adiposity, glucose intolerance, and insulin resistance [38]. In the current study and that of Davis et al, it was shown that gain or loss of function of adipocyte ERα significantly affects adipose tissue inflammation [11]. Similar results have been reported with increased adipose tissue 17β-estradiol production in males [14]. Furthermore, we have shown that skeletal muscle endogenous 17β-estradiol production decreases adiposity, adipose tissue inflammation, and hepatic lipid accumulation and normalizes hyperinsulinemia in males [20]. Hepatic ERα also plays an instrumental role in regulating gluconeogenesis, lipid metabolism, and hepatic insulin resistance in males [39, 40]. In this investigation, we observed that HFD consumption led to a reduction in adipose tissue ERα expression in male mice, but not in females, when compared to their respective LFD controls. Prior research in humans of both sexes has demonstrated an inverse association between adipose tissue ERα expression and visceral adiposity, along with a positive correlation with whole-body insulin sensitivity [12]. Therefore, the decreased ERα expression in male mice may be attributed to their greater adiposity and hyperinsulinemia relative to HFD-fed females. Further research is needed to elucidate the role of 17β-estradiol signaling in the regulation of metabolic health across sexes.

In conclusion, our findings highlight the complex role of adipose tissue ERα in regulating metabolic health and inflammation. This study provides evidence that enhancing ERα signaling in adipose tissue can have beneficial effects on obesity-related inflammation independent of changes in body weight or glucose metabolism. The sexual dimorphism observed in some outcomes underscores the importance of considering sex-specific effects in obesity research. This work contributes to our understanding of 17β-estradiol signaling in adipose tissue and its potential as a therapeutic target for obesity-related complications.

## Data Availability

Original data generated and analyzed during this study are included in this published article.

## Abbreviations

ANOVA: analysis of variance
DOX: doxycycline
ERα: estrogen receptor alpha
FFA: free fatty acid
GTT: glucose tolerance test
HFD: high-fat diet
KO: knockout
LC-MS: liquid chromatography–mass spectrometry
LFD: low-fat diet
qRT-PCR: quantitative real-time polymerase chain reaction
WT: wild-type
